# Salicylic Acid Co-Precipitation with Alginate via Supercritical Atomization for Cosmetic Applications

**DOI:** 10.3390/ma15217634

**Published:** 2022-10-30

**Authors:** Lucia Baldino, Ernesto Reverchon

**Affiliations:** Department of Industrial Engineering, University of Salerno, Via Giovanni Paolo II, 132, 84084 Fisciano, Italy

**Keywords:** alginate, salicylic acid, microparticles, supercritical assisted atomization, cosmetic applications

## Abstract

Alginate-based microparticles were produced via supercritical assisted atomization (SAA) with the aim of obtaining a biocompatible and low-cost carrier for the delivery of active compounds in cosmetic applications. Salicylic acid was selected as an active model compound, and it was co-precipitated with alginate via SAA, operating at 82 bar and 80 °C. In particular, the drug-to-polymer weight ratio was fixed at 1/4, whereas polymer concentration was varied from 5 to 20 mg/mL in the starting aqueous solution. Operating in this way, alginate-salicylic acid microparticles were characterized by a mean diameter of 0.72 ± 0.25 µm, and the active compound became amorphous after processing. A salicylic acid encapsulation efficiency close to 100% was reached, and the drug release time from the biopolymeric microparticles was prolonged up to nine times with respect to untreated salicylic acid powder.

## 1. Introduction

The cosmetic market is continuously expanding thanks to the growing attention of people to body’s health and aesthetic. In 2021, the global Italian cosmetic sector revenue exceeded 11.8 billion EUR; i.e., it increased by 9.9% compared with 2020, according to Cosmetic Study Center of Italy. A similar growing trend can be recorded in Europe and in the world, with skin-care products being at the top of the rankings [1]. However, the large use of cosmetics and personal care products is feeding environmental concerns due to presence of solid plastic particles that are synthetic and constituted of poorly biodegradable compounds [2,3,4,5]. For this reason, the replacement of synthetic polymers with natural and biodegradable ones and the use of sustainable technologies that can reduce water and energy consumption for the production of safe microparticles, encapsulating active principles, is the current demand of cosmetic industries [3].

Alginates are salts of alginic acid belonging to the family of linear block copolymers, constituted of α-L-guluronic acid and β-D-mannuronic acid residues linked by a glycosidic bond [6,7]. Sodium alginate is a low-cost, non-toxic, biodegradable anionic polysaccharide, generally obtained from brown seaweeds and some bacteria [6].

Salicylic acid is a non-steroidal anti-inflammatory drug, with antiseptic and analgesic effects. It is frequently used as an active compound in cosmetic products because of its topical effects [8].

Some authors tried to encapsulate salicylic acid in biopolymeric microcarriers, using different techniques [9,10,11], in the dermatology and cosmetology fields. In particular, microencapsulation is a technique where a biopolymeric compound is used to generate a physical shell around an active compound (e.g., active pharmaceutical ingredient, antioxidant, antimicrobial, antiaging, photoprotective, anti-inflammatory, etc.), to reduce the undesired action of external factors (e.g., temperature, light, pH, oxygen, etc.) and provide a controlled release of the active compound encapsulated in it [12,13]. The main techniques adopted for the production of microparticles encapsulating active compounds are [14,15,16]: emulsion polymerization, interfacial polymerization, coacervation/phase separation, solvent evaporation/extraction, sol-gel encapsulation, spray-drying, spray-cooling, and co-extrusion. However, these traditional methods suffer from various limits, mainly due to the difficulty in controlling particle size and size distribution, coalescence among particles, solvent residues, low drug encapsulation efficiency, and scale-up.

Supercritical assisted atomization (SAA) is an advanced version of the spray-drying technique that was proposed by Reverchon and co-workers [17,18,19] to produce biopolymeric microparticles. Specifically, a so-called gas-expanded liquid (GXL) [20] instead of a classical liquid solution is sprayed through an injector nozzle in a near-atmospheric pressure precipitation chamber. Operating in this way, the intrinsic cohesive forces, such as surface tension and viscosity, of the polymeric liquid solution are reduced because of the interposition of supercritical CO_2_ (SC-CO_2_) molecules among liquid molecules [21]. Therefore, the main advantages of this technique over the traditional ones are related to the possibility of obtaining smaller droplets exiting the injector that, after solvent evaporation induced by hot nitrogen, can be collected on a stainless-steel filter at the bottom of the precipitation chamber, in the form of microparticles [17,18,19,22]. Moreover, a good reproducibility in terms of particle size and size distribution can be assured, avoiding aggregation phenomena among particles [23]. Using this technique, several compounds have been co-precipitated [24,25], with the aim of improving the dissolution rate and thus the bioavailability of poorly water-soluble active pharmaceutical ingredients [26], controlling the drug release time and mechanism from the microcarrier [27], and producing microdevices for biomedical applications [28].

Therefore, the scope of this work is the production for the first time of alginate-based microparticles loaded with salicylic acid using SAA to control and to prolong salicylic acid dissolution time in order to obtain a longer effect for skin-care products.

## 2. Materials and Methods

### 2.1. Materials

Sodium alginate (powder; Mw ≈ 240 kDa) and salicylic acid (powder; Mw = 138.12 g/mol, purity ≥ 99%) were purchased from Sigma Aldrich (Milan, Italy). Carbon dioxide (CO_2_; 99.9% purity) was supplied by Morlando Group Srl (Sant’Antimo, NA, Italy) and nitrogen (N_2_; 99.9% purity) by SON (Naples, Italy). Distilled water was produced using a homemade lab-scale distiller.

### 2.2. Sodium Alginate-Salicylic Acid Solutions Preparation and SAA Apparatus Description

Sodium alginate was dissolved in water at concentrations ranging between 5 and 20 mg/mL at room temperature using a magnetic stirrer at 200 rpm, until a homogeneous solution was formed. Then, salicylic acid was added to the alginate aqueous solution (10 mg/mL), using a drug-to-polymer weight ratio (R) equal to 1/4.

The SAA apparatus consisted of two high-pressure pumps (mod. 305; Gilson, Cinisello Balsamo, MI, Italy) to deliver, respectively, the biopolymeric solution and liquid CO_2_ to a heated saturator. The saturator was a high-pressure vessel (25 cm^3^ internal volume) filled with stainless-steel perforated saddles, used to allow us to obtain a large contact surface between the biopolymeric solution and CO_2_, to form a gas-expanded liquid (GXL). The GXL was sprayed through a nozzle (80 μm internal diameter) into the precipitation vessel (3000 cm^3^ internal volume) operated at atmospheric pressure. A controlled flow of N_2_, previously heated using an electric heat exchanger (mod. CBEN 24G6; Watlow, Corsico, MI, Italy), was sent to the precipitator to induce droplet drying. A stainless-steel filter, located at the bottom of this chamber, was used to collect the dried microparticles, while the gaseous stream flowed out. The apparatus was completed by a separator for the recovery of the liquid solvent. SAA apparatus layout and further details about the experimental procedure are described in [18,22].

### 2.3. Characterization Techniques of Sodium Alginate-Salicylic Acid Microparticles

The morphology of the precipitated microparticles was observed using a field emission scanning electron microscope (FE-SEM; mod. LEO 1525; Carl Zeiss SMT AG, Oberkochen, Germany). Samples of the collected powder were deposited on an aluminum stub that was covered with a thin gold layer using a sputter coater (mod. 108 A; Agar Auto Sputter Coater; Stansted, United Kingdom) at 40 mA for 100 s; then, they were observed via FE-SEM.

The particle size (PS) and particle size distribution (PSD) of the powder were measured starting from SEM images and using Sigma Scan Pro image analysis software (version 5.0; Aspire Software International, Ashburn, VA, USA). Histograms representing the PSD were fitted using Microcal Origin Software (version 8.0; Origin Lab Corporation, Northampton, MA, USA). Approximately 300 particles were analyzed in the elaboration of each particle size distribution.

A differential scanning calorimetry (DSC) analysis was performed to determine the potential modifications of the characteristic temperatures of the biopolymer and the drug after mixing and supercritical processing. A 5 mg mass of each sample was analyzed using DSC (DSC 30 Mettler; Toledo, Spain) in a temperature range from 25 °C to 400 °C, using a heating rate of 10 °C/min; the inert gas was nitrogen, at a flow rate of 50 mL/min.

Salicylic acid encapsulation efficiency (EE%) and release rate from alginate microparticles were measured using a UV-Vis spectrophotometer (mod. Cary 60 UV-Vis; Agilent Technologies, Santa Clara, CA, USA) in triplicate. In particular, a microparticulate sample containing a theoretical amount of 50 ppm of salicylic acid was suspended in 1.5 mL of phosphate-buffered saline (PBS) at pH 7.4, selected as a release medium to simulate a cosmetic application. This sample was inserted in a 14,000 Da cut-off dialysis sack (Merck, Darmstadt, Germany) that was then immersed in 400 mL of PBS maintained at 37 °C and continuously stirred at 200 rpm. The drug absorbance was read at a wavelength (λ) of 310 nm under dark conditions. EE% was calculated using the following relationship:

EE% = [(effective loading)/(theoretical loading)] × 100

## 3. Results and Discussion

In the first part of the work, the feasibility of sodium alginate microparticle production via SAA was verified. The operating conditions selected for the process were a temperature of 80 °C and a pressure of 82 bar in the saturator, a temperature of 90 °C in the precipitation vessel, and a gas-to-liquid ratio (GLR) equal to 1.8 [19].

The name abbreviation of each set of experiments was: ALG01 (5 mg/mL sodium alginate), ALG02 (10 mg/mL sodium alginate), ALG03 (20 mg/mL sodium alginate), and ALG04 (10 mg/mL sodium alginate + salicylic acid, R = 1/4).

**Figure 1:**
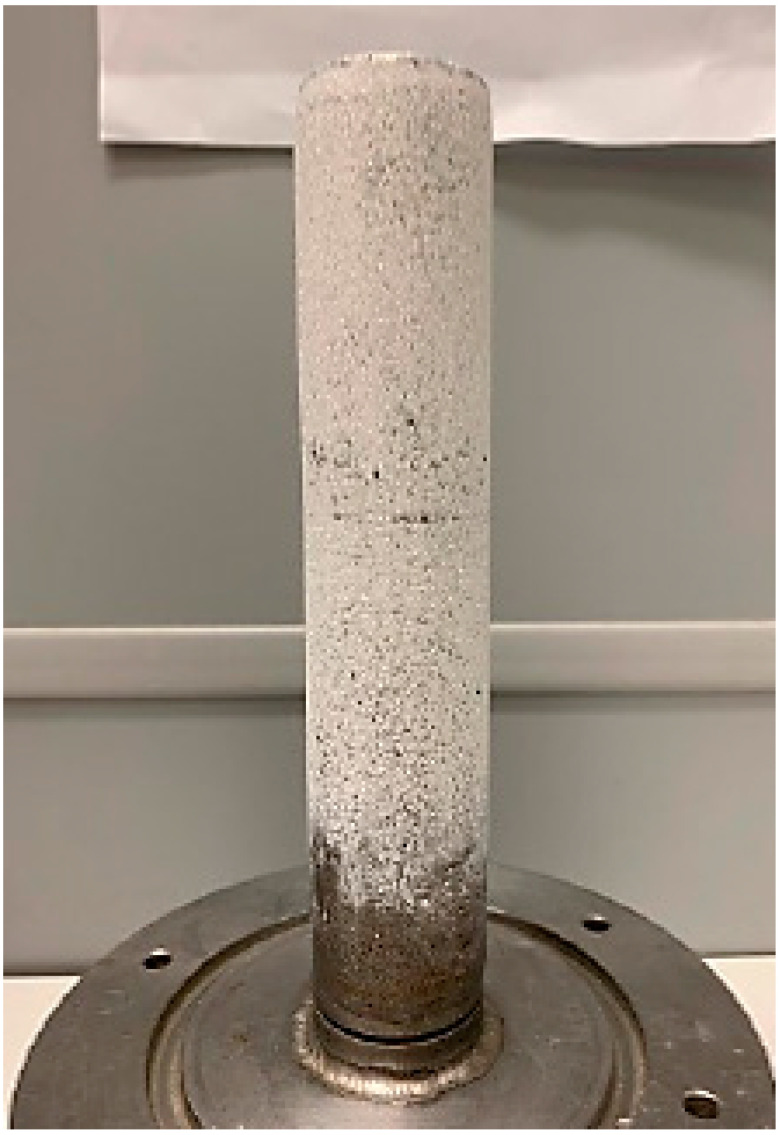
SAA filter covered by alginate powder.

A macroscopic evidence of the alginate powder produced via SAA is reported in Figure 1, where a photo of the collected white polymer particles is shown.

**Figure 2:**
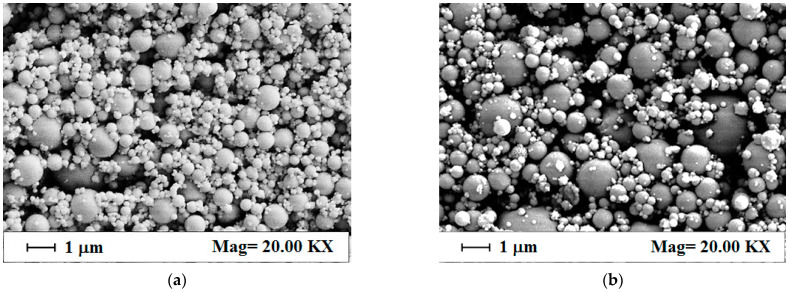

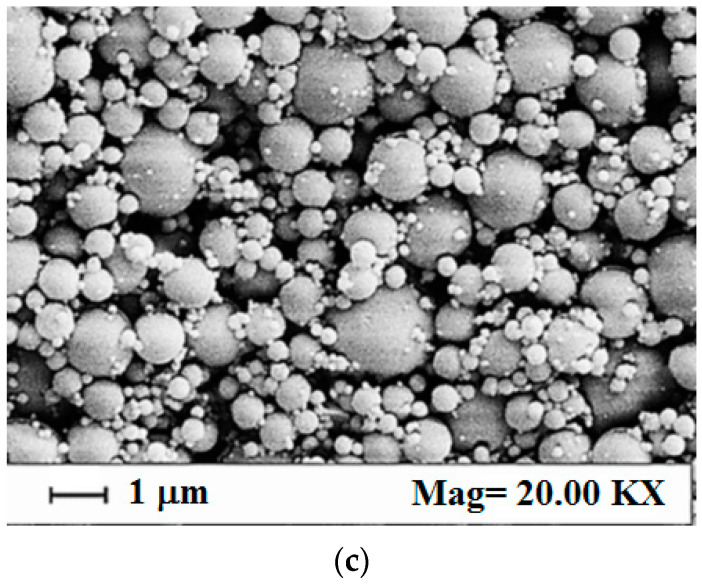
SEM images of sodium alginate microparticles produced via SAA at 80 °C, 82 bar, and GLR = 1.8: (**a**) 5 mg/mL, (**b**) 10 mg/mL, and (**c**) 20 mg/mL.

This observation was confirmed via the SEM images reported in Figure 2 that show for all the formulations tested the obtainment of spherical, non-coalescing, alginate-based microparticles. In particular, the increase in sodium alginate concentration in the starting aqueous solution, from 5 to 20 mg/mL, produced larger microparticles, as it can be qualitatively observed from Figure 2a to Figure 2c, where microparticles produced at 5, 10, and 20 mg/mL starting alginate concentrations are reported.

**Figure 3:**
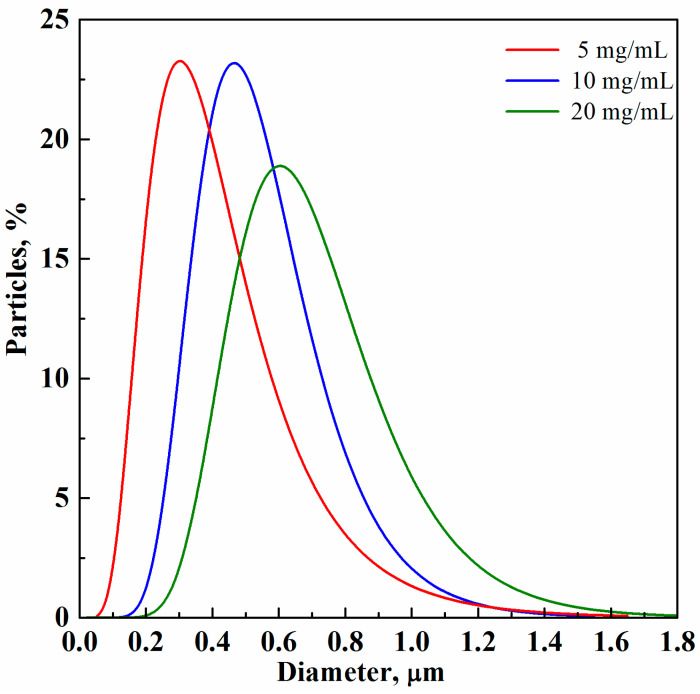
PSDs of sodium alginate microparticles produced via SAA at 80 °C, 82 bar, and GLR = 1.8.

SEM observations were quantitatively confirmed with the PSDs reported in Figure 3.

The alginate-based microparticle mean diameter varied from 0.30 ± 0.16 µm to 0.60 ± 0.24 µm when the biopolymer concentration increased from 5 to 20 mg/mL, respectively, as a consequence of the larger viscosity of the starting solution that produced an increase in the cohesive forces during the atomization process. More specifically, solution jet break-up is influenced by the competition between inertial forces (such as velocity at the injector nozzle) and cohesive forces (mainly viscosity and surface tension) that are computed in dimensionless numbers (e.g., Reynolds, Weber, and Ohnesorge numbers) generally used for the description of atomization processes. Therefore, even if a gas-expanded liquid characterized by reduced cohesive forces with respect to the corresponding liquid [20,21] was formed in the saturator before the injection step, the biopolymer concentration had a non-negligible effect on the final microparticle mean size; indeed, it influenced the obtainment of larger solution droplets that produced larger alginate microparticles after solvent evaporation [18,22].

Once we verified the possibility to produce alginate microparticles via SAA, salicylic acid was selected as the model compound to be loaded in it for potential cosmetic applications. An intermediate sodium alginate solution (i.e., 10 mg/mL biopolymer concentration) was used for the second part of the work, and the drug-to-biopolymer weight ratio was fixed at 1/4, with the aim of studying the interaction between the biopolymer and the drug, the drug encapsulation efficiency, and its release mechanism from the microparticles. SAA operating parameters were the same as those used in the previous experimental part.

**Figure 4:**
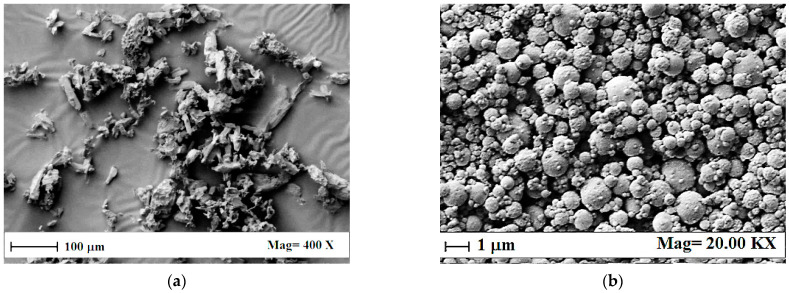
SEM images of (**a**) untreated salicylic acid powder and (**b**) sodium alginate-salicylic acid co-precipitated microparticles obtained via SAA at 80 °C, 82 bar, and GLR = 1.8.

The SEM images reported in Figure 4 show the morphology of untreated salicylic acid powder and sodium alginate-salicylic acid co-precipitated microparticles obtained via SAA. Untreated salicylic acid powder was characterized by a crystal-like shape, with mean dimensions between 10 and 100 µm (Figure 4a), whereas sodium alginate-salicylic acid co-precipitated microparticles showed a spherical morphology (Figure 4b), in line with the previous results obtained from sodium alginate alone. Moreover, since crystals were not observed in the microparticulate sample, this was an indirect information that all the drug was encapsulated into the biopolymeric microparticles.

**Figure 5:**
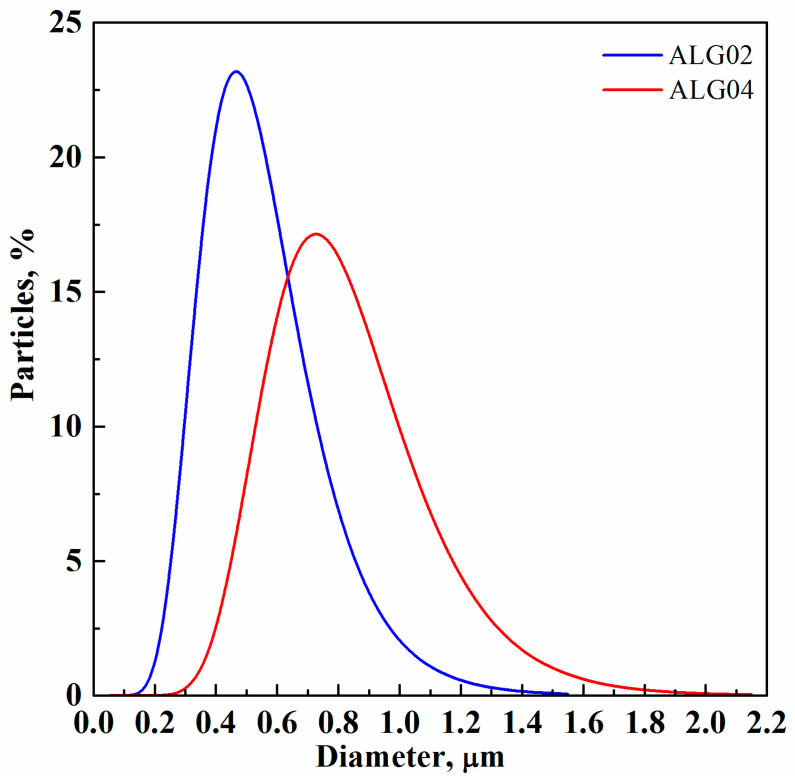
PSDs of sodium alginate microparticles and sodium alginate plus salicylic acid microparticles, produced via SAA at 80 °C, 82 bar, and GLR = 1.8.

The loaded microparticles showed a larger mean diameter with respect to the sodium alginate microparticles obtained at the same alginate concentration; in particular, the diameter changed from 0.46 ± 0.18 µm for sodium alginate microparticles to 0.72 ± 0.25 µm for sodium alginate plus salicylic acid microparticles. This result can be a consequence of the solution viscosity increase due to the presence of the drug [18,25,27], and this effect is also visible comparing the PSDs of these two different samples, as shown in Figure 5.

**Figure 6:**
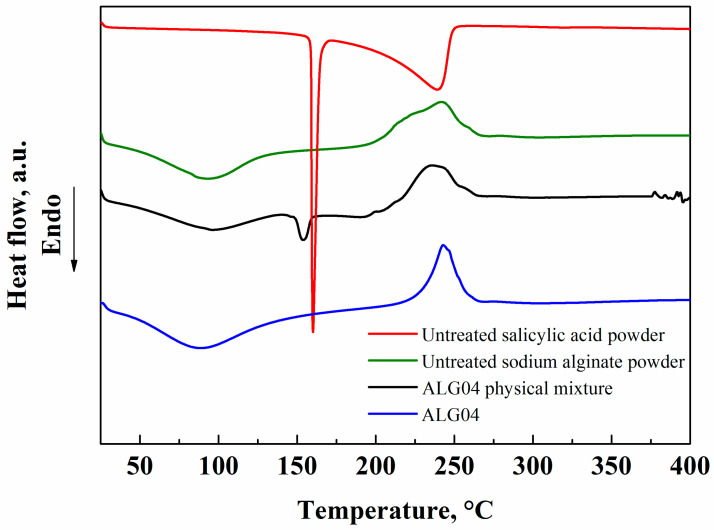
DSC analyses of sodium alginate (green) and salicylic acid (red) untreated powders, sodium alginate and salicylic acid untreated powders mixed in a 1/4 weight ratio (black), and loaded microparticles produced via SAA (blue).

Sodium alginate and salicylic acid untreated powders, sodium alginate and salicylic acid untreated powders mixed in a 1/4 weight ratio (physical mixture), and the loaded microparticles produced via SAA were analyzed with DSC to identify possible modifications in the characteristic temperatures of the two compounds after supercritical processing. The results are summarized in Figure 6.

Salicylic acid showed an endothermic (melting) peak at 160 °C, confirming its crystalline state [29]. Sodium alginate had a large endothermic peak around 100 °C, due to the humidity removal from the biopolymer [30], whereas the large peak detected at about 250 °C was due to the primary decomposition of the polymeric chains [30]. The physical mixture between sodium alginate and salicylic acid was characterized by all the temperature peaks identified in the thermograms of the single compounds, demonstrating that no interactions were formed among them. The loaded microparticles showed a thermogram similar to that of sodium alginate alone, suggesting that the drug became amorphous after supercritical processing. This behavior was already observed in other crystalline drugs [18,25], and it was due to the fast SC-CO_2_-assisted atomization that avoided the re-organization of the dissolved molecules in an ordinate form, together with the blockage of the dispersed particles of the drug in the polymeric matrix after solvent removal; both these contributions favored the amorphous state of the drug [22].

In order to verify the presence of the drug in the biopolymeric matrix, the salicylic acid encapsulation efficiency was measured following the procedure described in Section 2.3. A drug EE% equal to 98% was obtained, confirming the qualitative observation performed via SEM analyses.

**Figure 7:**
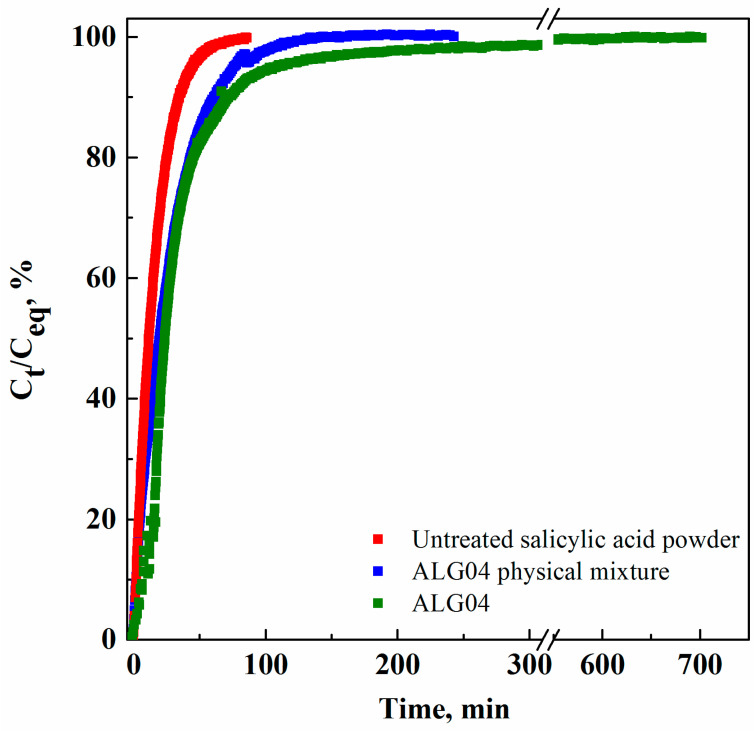
Salicylic acid release profiles from sodium alginate microparticles produced via SAA at 80 °C, 82 bar, and GLR = 1.8.

Moreover, drug release profiles were detected with a UV-Vis spectrophotometric analysis; the results are reported in Figure 7 as the ratios between the drug concentration measured at regular time intervals (C_t_) and the drug concentration at the equilibrium (C_eq_), against time.

The dissolution time of the untreated salicylic acid powder in PBS was around 80 min, whereas the release times of salicylic acid from the physical mixture and ALG04 microparticles were 250 and 700 min, respectively. Therefore, even if about 90% of the active principle was released in the first 100 min of the dissolution test, the presence of the biopolymeric carrier, in which the drug was co-precipitated, produced a salicylic acid release time increase of about nine times due to the mass transfer resistance opposed to the liquid diffusion in it. More specifically, sodium alginate tended first to swell in the liquid medium, and subsequently, the drug could be dissolved and released outside the carrier [31].

## 4. Conclusions

The SAA process confirmed its successful performance in producing alginate-based microparticles that were subsequently loaded with salicylic acid for potential cosmetic applications. Operating at 82 bar and 80 °C, spherical, non-coalescing microparticles were obtained, with a salicylic acid encapsulation efficiency close to 100%. Moreover, drug became amorphous after processing, and the drug release time from alginate microparticles was prolonged up to nine times with respect to untreated salicylic acid powder, evidencing the effect of the biopolymeric carrier on the active principle dissolution in the liquid medium.

## Data Availability

Not applicable.
